# Acoustophoretic Orientation of Red Blood Cells for Diagnosis of Red Cell Health and Pathology

**DOI:** 10.1038/s41598-018-33411-0

**Published:** 2018-10-24

**Authors:** Laura G. Rico, Jordi Juncà, Mike D. Ward, Jolene A. Bradford, Jorge Bardina, Jordi Petriz

**Affiliations:** 1 Functional Cytomics Group, Institut de Recerca contra la Leucèmia Josep Carreras (IJC) Campus ICO-Germans Trias i Pujol, Universitat Autònoma de Barcelona (UAB), 08916 Badalona, Spain; 2 Institut Català d’Oncologia, Hospital Germans Trias i Pujol (HGTiP), Badalona, Spain; 3 Thermo Fisher Scientific, Eugene, Oregon USA

## Abstract

Distortions of the normal bi-concave disc shape for red blood cells (RBCs) appear in a number of pathologies resulting from defects in cell membrane skeletal architecture, erythrocyte ageing, and mechanical damage. We present here the potential of acoustic cytometry for developing new approaches to light-scattering based evaluation of red blood cell disorders and of the effects of storage and ageing on changes or damage to RBCs membranes. These approaches could be used to immediately evaluate the quality of erythrocytes prior to blood donation and following transfusion. They could also be applied to studying RBC health in diseases and other pathologies, such as artificial heart valve hemolysis, thermal damage or osmotic fragility. Abnormal distributions of erythrocytes can typically be detected after just 30 to 45 seconds of acquisition time using 1–2 µL starting blood volumes.

## Introduction

The precision of flow cytometry measurements for non-spherical cells such as sperm cells and red blood cells is affected by the orientation of cells as they pass through a light source. This is especially true for light scatter measurements, where nearly identically shaped cells can have significantly different scatter signals owing to the variation in the cross section of each individual cell facing a focused laser source. For red blood cells, this variation is further complicated by secondary light scattering effects arising from their unusual bi-concave disc shape^1^.

In 1977, Mack Fulwyler^2^ demonstrated hydrodynamic cell orientation with chicken erythrocytes and he suggested that controlling the orientation of non-spherical particles would improve precision of analysis in flow cytometry. Following the lead of Stovel and Herzenberg^3^ and later Johnson and Pinkel^4^, shaped nozzles designed to orient cells by introducing asymmetries into the velocity flow field of hydrodynamic sheath systems, have since been implemented to improve sex sorting of sperm in the dairy industry. Asymmetric acoustic standing wave fields can also orient non-spherical particles and exploiting this effect for improving flow cytometry measurements has similarly been suggested^5^. Commercial acoustic flow cytometers combine hydrodynamic focusing with acoustic focusing by using an ultrasonically resonant capillary to inject sample into the instrument’s sheath flow. The resonant capillary, similar to the device first described by Goddard and Kaduchak^6^, focuses cells into a single line in the center of the capillary according to the cells’ intrinsic properties of size, density, and compressibility relative to the media in which they are suspended. The acoustic focus of suspended particles or cells prior to injection is typically used to achieve high precision alignment of cells at sample flow rates up to 10-fold greater than with conventional flow cytometers using hydrodynamic focusing only. The acoustically resonant injection capillary has a circular interior cross section, but the acoustic forces are not radially symmetric, and this field asymmetry is responsible for the acoustic orientation of non-spherical cells or particles^7^. The light-scattering signal from a red blood cell depends on a complex set of numerous factors related to both the optical excitation and collection parameters of the instrument and the blood cell size, shape, orientation, and internal structure and composition^1,8^. Measurement of red blood cell size and hemoglobin content using two angles of scatter in a flow cytometer was reported by Tycko *et al*. in 1985^9^ but the complexity of native shaped red blood cell scatter signals required this analysis to be done with osmotically sphered erythrocytes, a practice commercialized in some blood analyzers using a procedure described by Kim^10^. Gilev *et al*.^11^ reported analysis of multiple parameters for native shaped red blood cells using their scanning flow cytometer^8^, an instrument capable of measuring single cell scatter signals over a range of 15 to 55 degrees by collecting specific angles of incidence in time as the cell transits a laser source. The data generated is termed an “indicatrix”, a plot of scatter intensity vs. angle for an individual cell passing through the system.

For the results presented here, the data is collected over conventional fixed integrated angles of low angle forward scatter and orthogonal side scatter. The role of the acoustic orientation effects are not yet fully understood as they pertain to the observed differences in the various populations tested. These differences are however indicative of each of the conditions tested, and they show promise that this method could be developed for reagent free study of red blood cell (RBC) health and pathology.

## Results and Discussion

### Acoustic orientation of red blood cells

**Figure 1:**
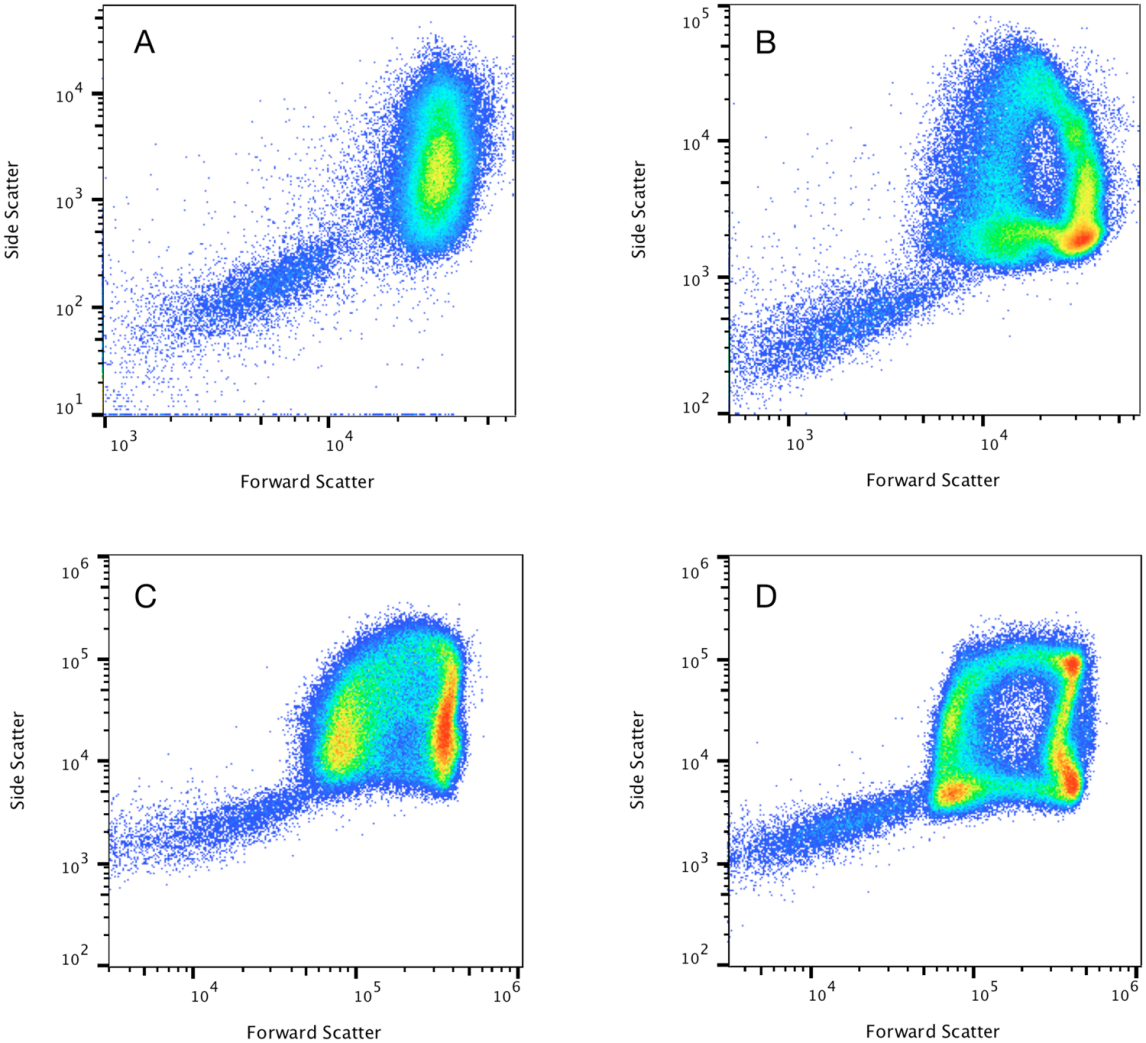
Comparative effect of hydrodynamic focusing and acoustic focusing using unlysed diluted blood from a healthy subject. Representative population distributions for diluted whole blood showing red blood cells (RBCs) and platelets (PTLs) obtained with hydrodynamic instrument (**A**) (displayed in dotplot **A**) with hydrodynamic instrument (**B**) (displayed in dotplot **B**) with acoustic-assisted focusing turned off (displayed in dotplot **C**) and with acoustic focusing turned on (displayed in dotplot **D**). Forward scatter vs. side scatter dotplots display well-defined characteristic arch-shaped populations of erythrocytes only when acoustic focusing is turned on.

After noticing unusual arch-shaped patterns for RBCs on forward vs. side scatter plots of diluted whole blood collected on the acoustic focusing cytometer, experiments were run to determine how unique these patterns are to this instrument and whether the acoustic focusing in the sample injector was playing a role. Figure 1 shows the forward vs. side scatter patterns of red blood cells when analyzed on two different hydrodynamic only focusing flow cytometers, and on the Attune NxT Flow Cytometer which combines both hydrodynamic and acoustic focusing. Hydrodynamically focused instrument A showed a single diffuse RBC population, having an overall side scatter distribution covering an order of magnitude in intensity and a forward scatter distribution approximately 3 times smaller. Hydrodynamically focused instrument B had a broader distribution in forward scatter with more distinct populations characteristic of bi-variate distributions observed by others^12^. The difference in patterns for the hydrodynamically focused cytometers is likely a combination of differing scatter collection angles and the degree to which the hydrodynamic focus of each instrument orients the cells. Forward scatter signal should be particularly sensitive to differences in collection angle, as the smallest angles, up to about 3–5 degrees, are expected to have the largest contribution to signal, with signal contribution at these small angles significantly affected by the cell orientation^13^. The patterns observed on hydrodynamic focusing instrument B were more similar to the patterns observed on the acoustic focusing flow cytometer, with greatest similarity to those taken when the same cytometer had acoustics turned off with hydrodynamic focus only. These experiments show that the patterns observed on the acoustic cytometer are a combination of the instrument’s hydrodynamic focus and light scatter collection angles, combined with an acoustic effect. If the cells are identically positioned and oriented in the illuminating laser, it is expected that cells of similar morphology will have similar scatter signals. The data shows multiple distinct populations however, suggesting that the red blood cells are not all uniformly oriented with respect to the laser. The acoustic field does not completely orient all cells on one axis and it is also possible that given that acoustic focus occurs prior to hydrodynamic focus, the red blood cells exiting the sample injection capillary have an opportunity to tumble and alter their orientation in the hydrodynamic focusing flow field prior to passing through the laser beam.

### Erythrocytes patterns and pathology

Scatter plots for freshly drawn blood from healthy donor volunteers were shown to have well-defined arch-shaped distributions of erythrocytes only when acoustic focusing is turned on (Supplementary Fig. I). Some subjects with anemia were shown to have a population of normocytes in addition to the predominant macrocytic or microcytic population, which in combination exhibited different arch-shaped distributions when compared with freshly drawn blood from healthy donor volunteers (Supplementary Fig. II).

An arch shaped pattern is discernable with the acoustic field turned off (Fig. 1C) and in the data for hydrodynamic only focusing instrument B (Fig. 1B), indicating that the pattern is not solely due to the acoustic orientation effect. While adjusting laser alignment and or changing light collection angles will affect the pattern, the comparative data with these parameters fixed and the acoustic field turned on (Fig. 1C) or off (Fig. 1D) shows the most compelling demonstration of the impact of the acoustic orientation effect.

With the acoustic field turned on, the arch shaped pattern becomes less diffuse (Supplementary Movie I), suggesting a less random orientation than for hydrodynamic focus alone. While it is expected that cells may tumble after acoustic focus, any tumbling should be more deterministic if cells enter the sheath flow with more uniform orientations to begin with. If this is the case, the range of possible orientations may be more limited which could in turn explain the tighter definition seen in the patterns.

For fresh healthy blood samples with acoustics turned on, the scatter patterns manifest a well-defined gap in the center at intermediate intensities of forward and side scatter (Fig. 1D). For samples expected to have red blood cell shapes deviant from fresh healthy cells, additional populations appeared in this gap. We began to prospectively screen individuals with pathological and non-pathological conditions and then explored how these RBC distributions might show patterns to differentiate pathognomonic signs characteristic of different red blood cell disorders (Supplementary Movie II). RBCs have membranes with a complex structure, and loss of cell surface area/volume ratio leads to shape changes that make them prone to increased osmotic fragility (Supplementary Movie III), and the formation of morphologically abnormal erythrocytes which are susceptible to destruction by macrophages^14^.

### Defects in Erythrocyte Membrane Skeletal Architecture

We studied RBC disease with defects in cell membrane skeletal architecture and remodeling of the cell membrane as a consequence of erythrocyte ageing, which can result in alterations of cell deformability, elasticity or viscosity. Currently available methods to study such parameters on RBCs include rotational viscometers^15^ and ektacytometers^16,17^. However, these techniques do not take into consideration the heterogeneity or size differences within the sample population of erythrocytes. Hereditary spherocytosis (HS) is one of these diseases with defects in the cell membrane. It results from mutations in genes encoding for structural proteins with a key role in mechanical stability, and is characterized by the presence of spherical-shaped erythrocytes and the occurrence of hemolytic anemia not caused by an autoimmune reaction.

Flow cytometry has become a very useful tool in the screening of membranopathies and different approaches have been described that are intended to calculate the osmotic fragility of diseased RBCs or to measure eosin-5′-maleimide (EMA) binding^18^. Mean sphered corpuscular volume (MCV) values obtained from hematology analyzers have also been evaluated to predict HS^19^. However, HS can be easily misdiagnosed and may occasionally be mistaken for Gilbert syndrome^20^.

**Figure 2:**
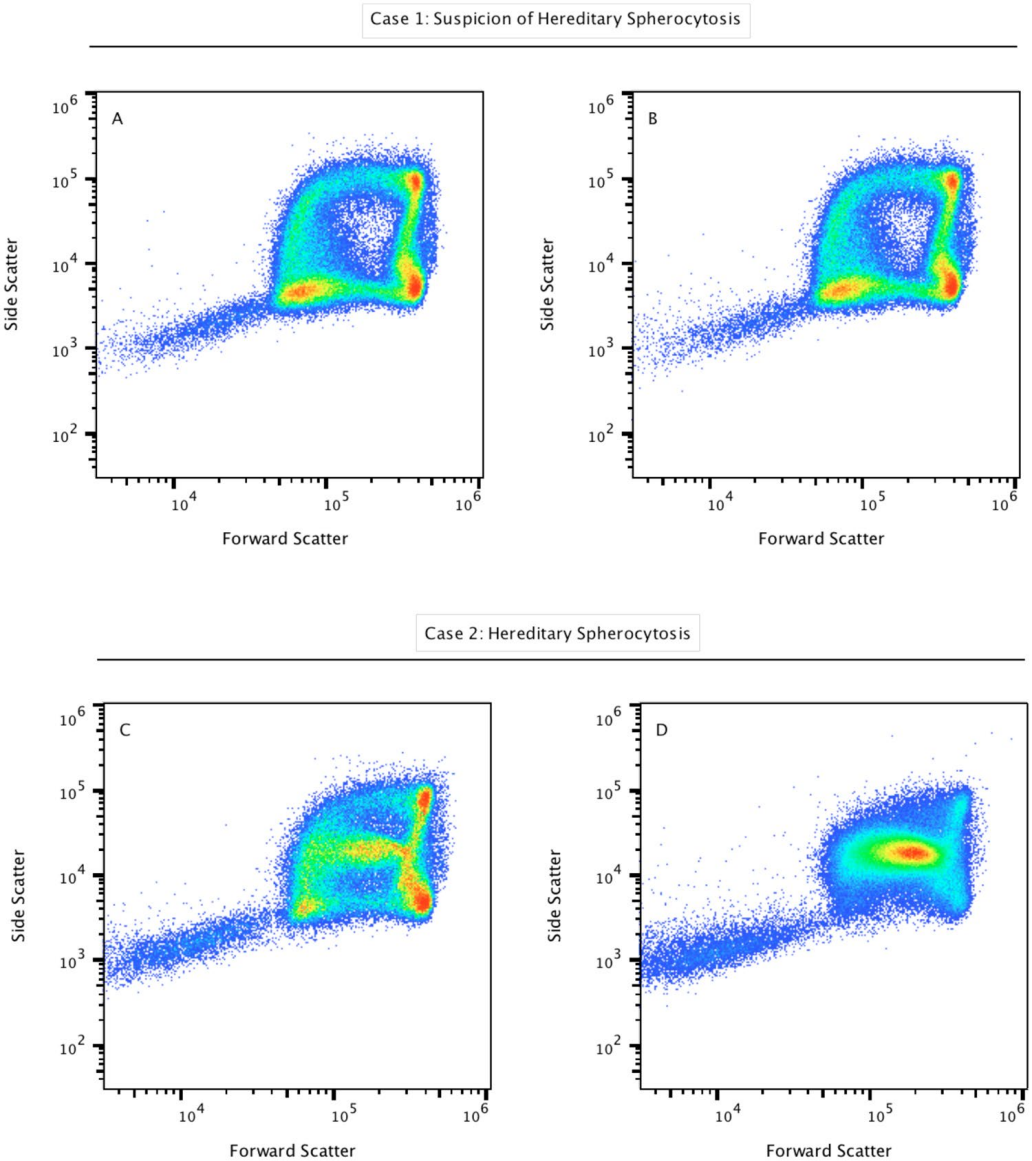
Testing for the clinical suspicion of hereditary spherocytosis. Light-scatter properties of red blood cells obtained from a healthy subject with suspicion of hereditary spherocytosis (upper panel), and comparisons with the scatter properties of a patient with confirmed hereditary spherocytosis (lower panel). Samples were processed in duplicates and were maintained at room temperature (**A** and **C**) and incubated at 37 °C for 15 min (**B** and **D**). Representative results for the abnormal distributions of erythrocytes from a subject with hereditary spherocytosis are shown in the lower panel. As can be seen in these dotplots, the presence of spherocytes provides a different scatter profile when compared with healthy controls, showing spherocytes with intermediate forward and side scatter properties, in figures **C** and **D,** respectively. Spherocytes represent the main population in **D** as a consequence of osmotic balance and permeability disruption.

As shown in Figure 2, hereditary spherocytosis subject samples demonstrate a clearly different pattern based on the light-scatter measurements. Figure 2A shows the representative shape distribution of erythrocytes in a patient with suspicion of hereditary spherocytosis, prepared at room temperature. Figure 2B shows the same sample incubated at 37 °C for 15 minutes. Representative results for the abnormal distributions of erythrocytes from a patient with confirmed hereditary spherocytosis are shown in Figure 2C,D. As can be seen in these dotplots, the presence of spherocytes produces scatter patterns with a distinct additional population suggesting that spherocytes show intermediate forward and side scatter intensity in the gap region present in healthy controls (Fig. 2C,D). Spherocytes represent the main population in Figure 2D, where the cell suspension was incubated for 15 min at 37 °C, supporting the idea that thermal damage is also associated with spherocytosis^21^. This simple experiment suggests that spherocytes are more sensitive to cell membrane damage caused by the increased gas exchange, alterations of steady-state glycolysis, and disruption of the osmotic balance and permeability, when cells are incubated at 37 °C.

### Storage and mechanically-induced damage of red blood cells

**Figure 3:**
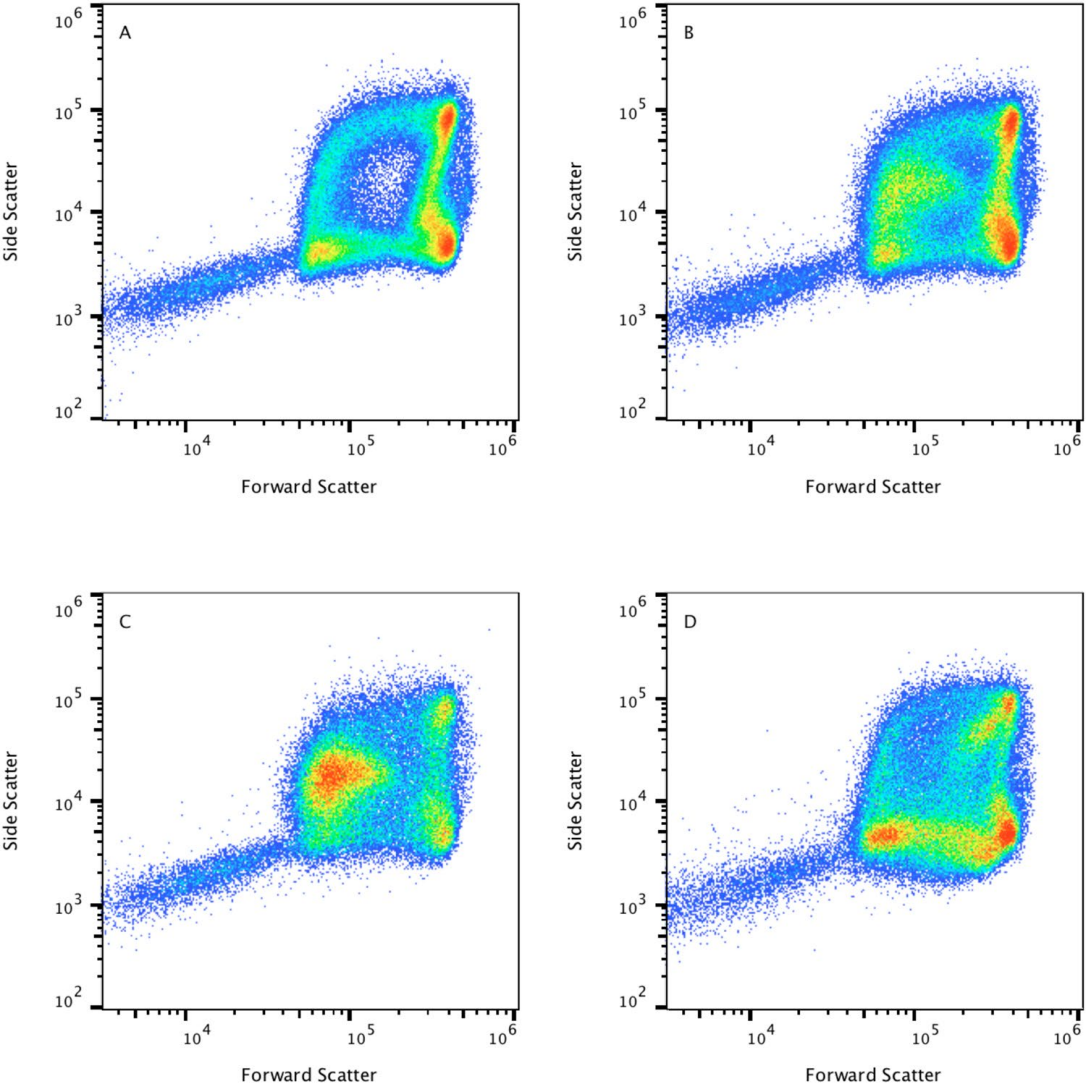
Changes in erythrocyte light-scatter properties resulting from storage, ageing, and mechanical damage showing erythrocyte fragility and, propensity to hemolyze. Scatter light properties of normal blood after venipuncture (**A**) 24 hours after venipuncture (**B**) transfused circulating erythrocytes (**C**), and fragmented red blood cells or schistocytes in peripheral blood (**D**). Spherocyte-like cells with intermediate to low forward scatter properties are shown in **B** and **C** and are absent in **A**. Schistocytes are displayed in **D** showing low relative forward and side scatter properties, as a consequence of red cells fragmenting in a patient with an artificial heart valve.

We were also interested in changes in erythrocyte shape resulting from ageing, storage, and mechanical damage showing erythrocyte fragility and propensity to hemolyze. In Figure 3 we analyzed the effect of storage at room temperature, monitoring the scatter-light patterns immediately after venipuncture (Fig. 3A) and again at 24 h (Fig. 3B). In addition, we studied the presence of circulating transfused erythrocytes after transfusion of stored RBCs (Fig. 3C) as well as the increased presence of circulating fragmented RBCs, or schistocytes in peripheral blood (Fig. 3D). It has been described that erythrocytes become progressively rigid and incapable of being flexible, less deformable and dense with age^22^. At the end of erythrocyte lifespan, the normal discoid biconcave shape will turn into a spherocyte or into a stomatocyte.

**Figure 4:**
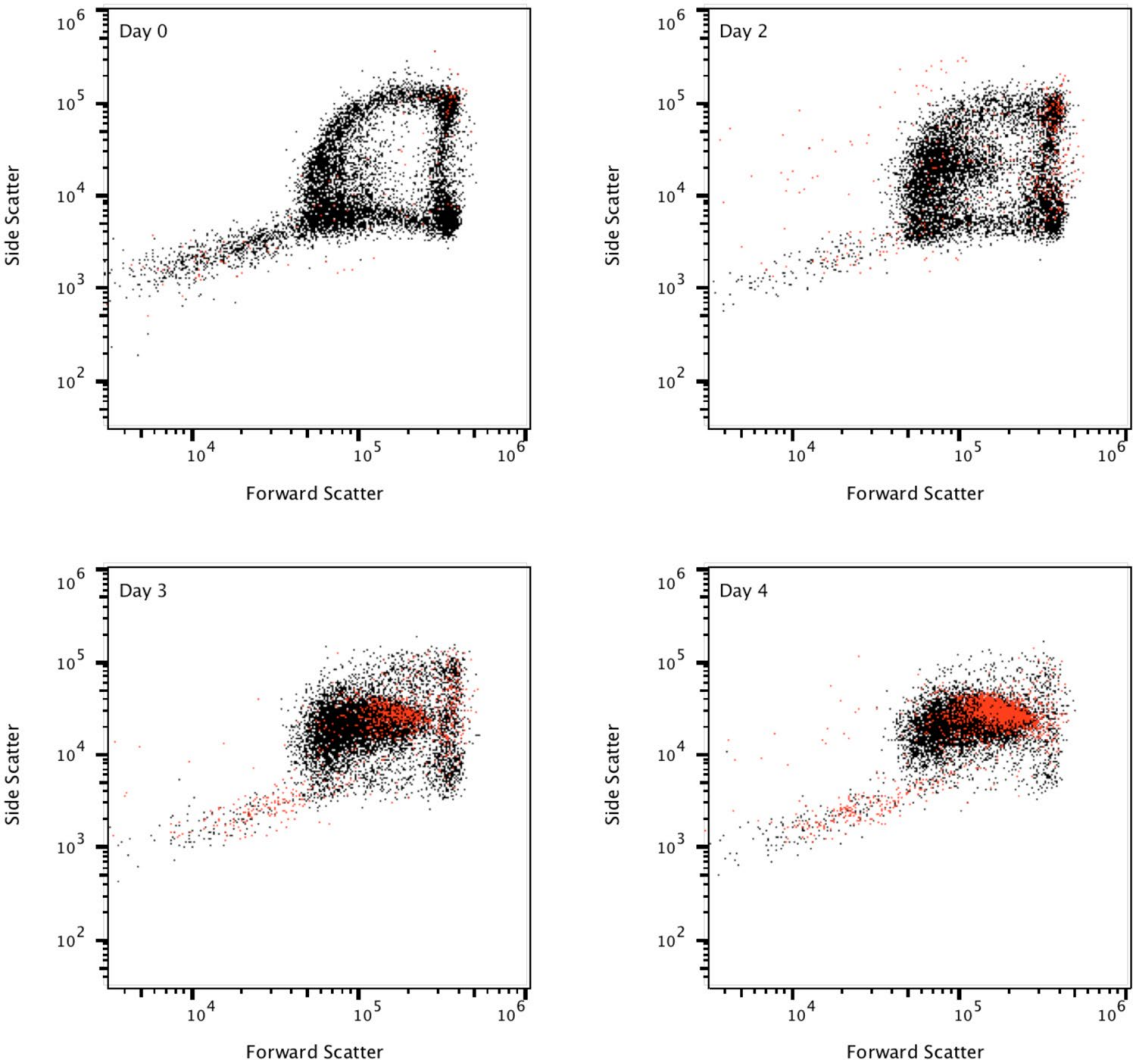
Stimulation of erythrocyte phosphatidylserine (PS) exposure by aging. This experiment was performed over 5 consecutive days. EDTA anticoagulated whole blood from a healthy subject was stored at room temperature and PS exposure was detected with PE-annexin V. Spherocytes appear to age faster among red blood cells, starting to show collapse and loss of membrane integrity on day 3 to 4. Note that events collected appear to be less than 100,000 as a consequence of dotplot overlaying. Red dots represent PE-annexin V positive events.

We sought to track this process by using fluorescent Annexin V reagent to illustrate an example of how the simple protocols described can be expanded to further study red blood cells. This test shows programmed red cell death on an individual cell by cell basis. This programmed death prevents hemolysis and inflammation (Fig. 4). The arch-shaped distribution is heavily affected with aging, even when freshly drawn blood obtained from healthy donor volunteers is used. Microscopic examination of freshly drawn blood from a healthy donor blood smear detected a variety of morphological alterations in red blood cells induced in the cell membrane as a consequence of accelerated ageing for a period of three days. Red blood cells acquired morphologic features of spiculated spherocytes (Supplementary Fig. III).

### RBC volume measurement

**Figure 5:**
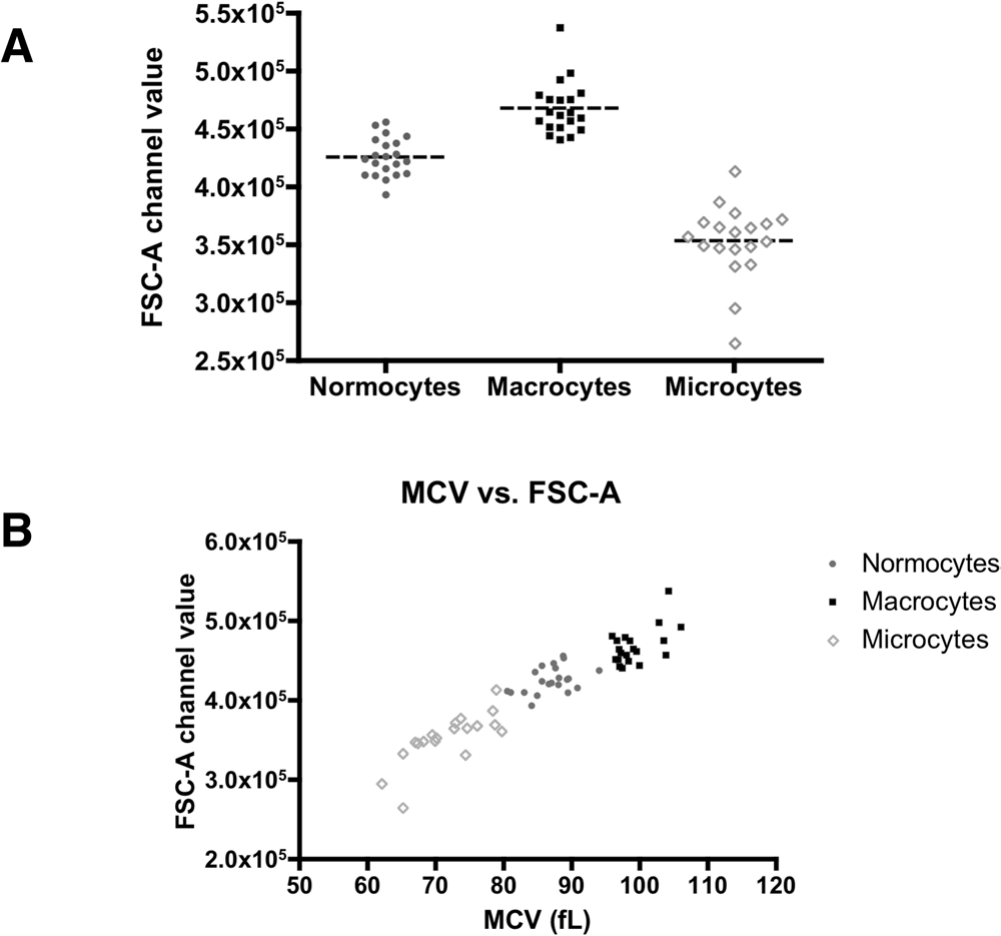
Red blood cell size as measured using the forward scatter parameter on the acoustic focusing cytometer. This experiment was performed on normocytic, macrocytic and microcytic human peripheral blood specimens (n = 20 per group). The FSC-A parameter value was collected according to the region displayed in Supplementary Fig. II, and used to display normocytic, macrocytic and microcytic red blood cells (**A**). The data were analyzed using the Kruskal-Wallis test with 95% coincidence intervals. Statistically significant differences were observed (Kuskal-Wallis statistic = 49.37, p-value = 0.0001). Automated hematology analyzers methods were also used to display directly-measured parameters on the MCV red cell index, with the mean FSC-A parameter value on the acoustic flow cytometer (**B**).

Finally, we used the acoustic flow cytometry data to measure cell size in comparison with automated hematology analyzer measurements of the mean corpuscular volume (MCV) or cell size, a red cell index used to help diagnose red cell pathologies. The mean forward scatter-area (FSC-A) parameter showed statistically significant differences in the size of red blood cells that corresponded with the clinical classification of them into normocytes, macrocytes and microcytes, with normal, high, and low MCV, respectively (Fig. 5).

Here we show the potential of acoustic flow cytometers for developing new approaches using light-scattering to evaluate the effects of storage and ageing on changes or damage to RBC membranes, or to immediately evaluate the quality of erythrocytes following blood donation. Such approaches may also be useful in other research areas, including the study of heart valve hemolysis as a consequence of mechanical trauma in patients with artificial valves.

## Materials and Methods

### Samples

EDTA-anticoagulated human blood samples were obtained from samples taken for clinical testing purposes not related to this work. Fresh blood from healthy donors and donors with microcytic and macrocytic anemia, blood from hereditary spherocytosis subjects, ageing and transfused blood (1 to 5 days), and blood from a subject with an artificial heart valve were used in this study. The stimulation of erythrocyte phosphatidylserine (PS) exposure by aging was performed using EDTA anticoagulated whole blood stored at room temperature on 5 consecutive days. May Grünwald-Giemsa (Merck) staining was used for the morphological assessment of the cells (Supplementary Fig. III). All procedures were performed in accordance with the internal protocols of our laboratory, which were authorized by the HGTiP’s Ethical Committee, in accordance with current Spanish legislation, by the Departament de Medi Ambient i Habitatge (file #1899) of the Autonomous Government of Catalonia (Generalitat de Catalunya). Blood samples were obtained from patients with their written informed consent according to the protocol approved by the Ethical Committee of the Germans Trias i Pujol Hospital.

### Materials

Hanks’ Balanced Salt Solution (HBSS), no calcium, no magnesium, no phenol red, was obtained from Capricorn Scientific GmbH (Germany). All materials related to flow cytometry, such as focusing fluid, shutdown and wash solutions, Attune™ performance tracking beads, and PE-Annexin V, were obtained from Thermo Fisher Scientific.

### Flow cytometry

Samples were acquired on two hydrodynamic focusing cytometers and on the Invitrogen™ Attune™ NxT acoustic focusing cytometer (Thermo Fisher Scientific). Briefly, 2 µL of blood were diluted 1:500 in HBSS to a total volume of 1000 µL, and immediately acquired for light-scattering measurements. After gently mixing for a few seconds to prepare homogeneous cell suspension, samples were immediately acquired.

Acquisition was performed at the lowest available sample rates: 12.5, and 25 µL/s to minimize coincident events on each hydrodynamically focused cytometer and on the Attune NxT respectively. The comparative effect of the flow speed is shown in Supplementary Fig. IV. Sample acquisition times ranged from 30 to 45 seconds depending upon erythrocyte counts. Acquisition was stopped when approximately 100,000 total cells were collected. Forward scatter and side scatter are shown in logarithmic scale. Contour plots were used to better discriminate differences in forward scatter and used for size comparison purposes. Specifically, the mean FSC-A parameter value was collected using the region displayed in Supplementary Fig. II, for normocytic, macrocytic and microcytic comparisons. Annexin V was incubated for 20 min at room temperature in binding buffer (10 mM HEPES, pH 7.4, 2.5 mM CaCl_2_, 140 mM NaCl).

### Data analysis

FlowJo software v10.4.2 was used for the analysis of flow cytometry data. Single FCS files obtained from normocytes (n = 10) and spherocytes (n = 10) studies were concatenated and downsampled in order to visualize 100,000 events. For comparative analysis, the new concatenated file, was used to display normocytes, spherocytes, and the whole merged population (Supplementary Fig. V).

## Electronic supplementary material

Supplementary Information

Movie 1

Movie 2

Movie 3
