# Supplementary material for: Acoustophoretic Orientation of Red Blood Cells for Diagnosis of Red Cell Health and Pathology
Source: Sci Rep. 2018 Oct 24;8:15705. doi: 10.1038/s41598-018-33411-0 (PMC6200821; doi:10.1038/s41598-018-33411-0)
Supplement: Supplementary file 1 — Supplementary Information [file 41598_2018_33411_MOESM1_ESM.doc]

***TITLE:***

**Acoustophoretic Orientation of Red Blood Cells for Diagnosis of Red Cell Health and Pathology**

***AUTHORS:***

Laura G. Rico^1^, Jordi Juncà^1,2^, Mike D. Ward^3^, Jolene A. Bradford^3^ , Jorge Bardina^1^, and Jordi Petriz^1^

^1^ Functional Cytomics Group [Institut de Recerca contra la Leucèmia Josep Carreras], IJC Campus ICO-Germans Trias i Pujol, Institut Germans Trias i Pujol (IGTP), [Universitat Autònoma de Barcelona], UAB, 08916 Badalona, Spain

^2^ Institut Català d’Oncologia, Hospital Germans Trias i Pujol (HGTiP), Badalona, Spain

^3^ Thermo Fisher Scientific, Eugene, Oregon, USA

***RUNNING TITLE:***

Acoustic Cytometry of Red Blood Cells

***KEYWORDS:***

Red blood cells, erythrocytes, acoustic focusing cytometry, light scattering, hereditary spherocytosis, hemoglobin, cardiac valves, ageing, transfusion, blood banking, hemolysis.

***CORRESPONDING AUTHOR:***

Jordi Petriz, PhD

Josep Carreras Leukaemia Research Institute

Crta. de Can Ruti, Camí de les Escoles s/n. Edifici IJC

08916 Badalona (Barcelona), Spain

Email: [jpetriz@carrerasresearch.org](mailto:jpetriz@carrerasresearch.org)

***CONFLICT OF INTEREST:***

M.D.W. and J.B. work for ThermoFisher Scientific, which is in the business of selling flow cytometers and flow cytometry reagents.

***AUTHOR CONTRIBUTION STATEMENT:***

L.G.R., J.B. and J.P. performed the experiments. J.J., M.D.W., J.A.B., and J.P performed data interpretation, and drafted the manuscript. J.P. conceived of the study, participated in its design, conducted most experiments, and revised the manuscript.

Supplementary Figure I


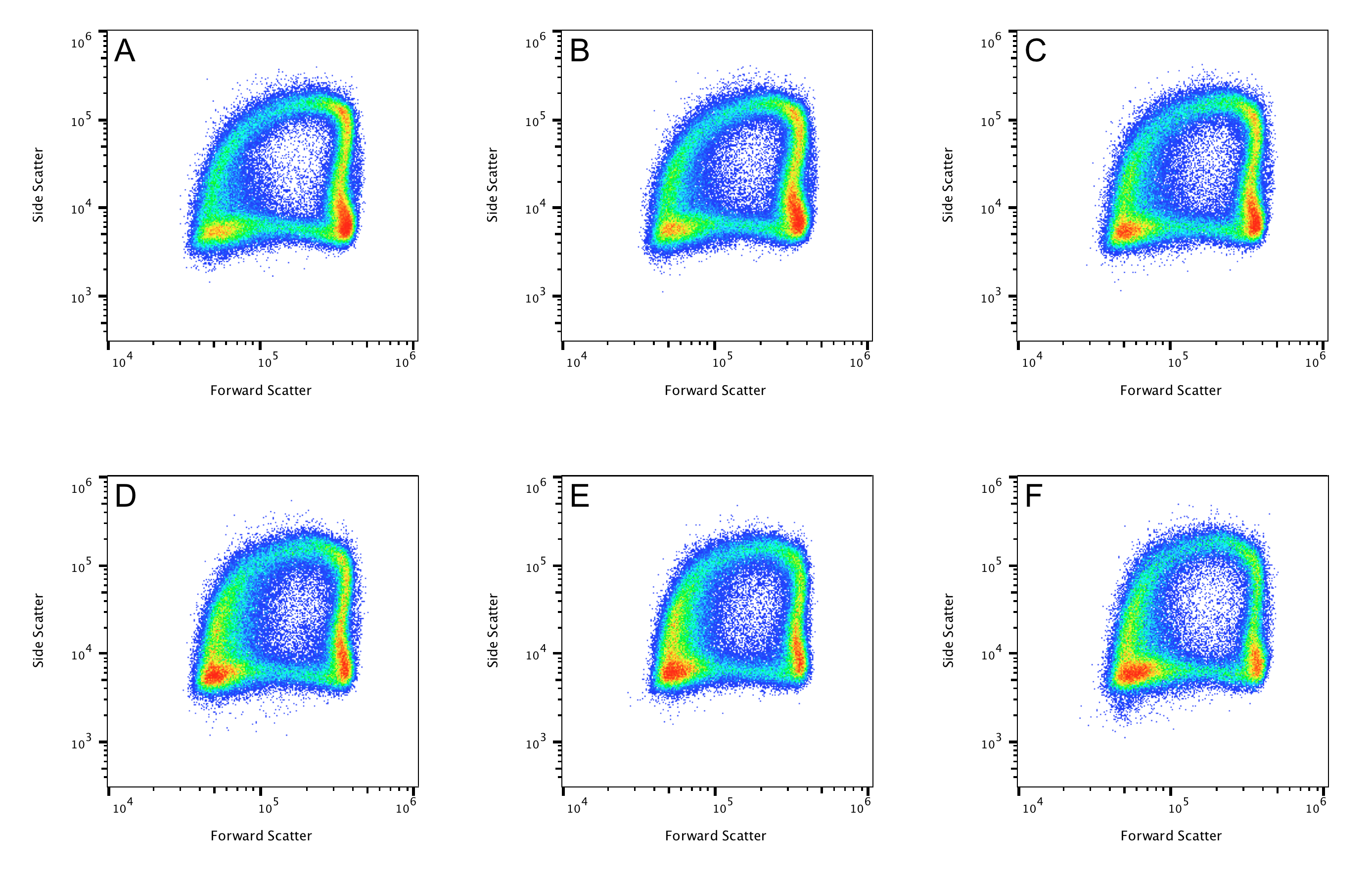


**Light-scatter properties of red blood cells obtained from six different healthy donors.** Samples were diluted as described and acquired at 12.5µL/min sample speed. Representative results for the arch-shaped distributions of erythrocytes are shown from A to F.

Supplementary Figure II


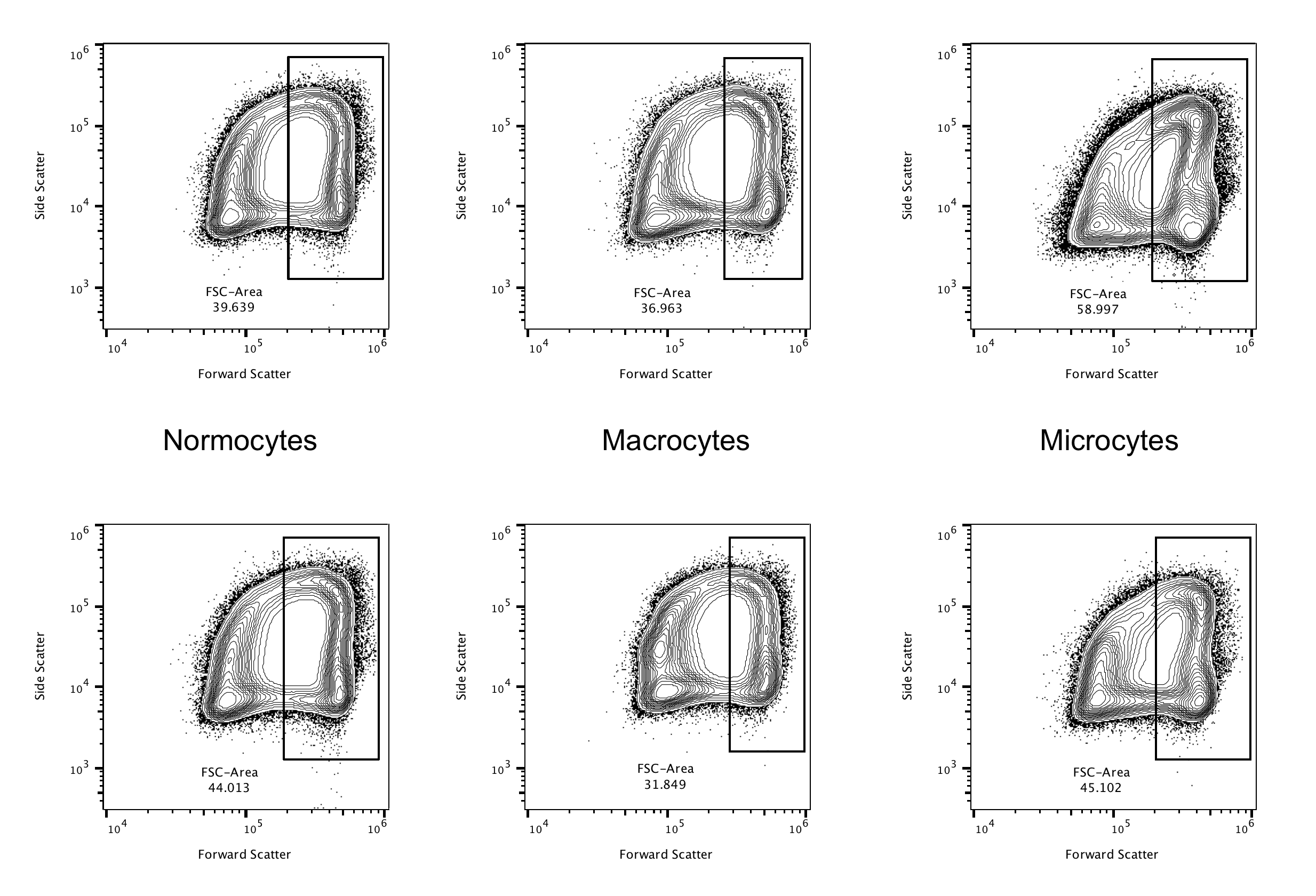


**Representative contour-plots from normocytic (normal MCV), macrocytic (increased MCV) and microcytic (decreased MCV) freshly drawn blood samples.** Contour plots were used to better discriminate differences in forward scatter and used for size comparison purposes. Two representative specimens from each group are displayed. The statistics in the region represents percentage of the gate. Average size comparisons of normocytic, macrocytic and microcytic cells used the mean FSC-A parameter obtained from the gate displayed. Note that gate position was adjusted to the lowest contour level on the same plot.

Supplementary Figure III

A)


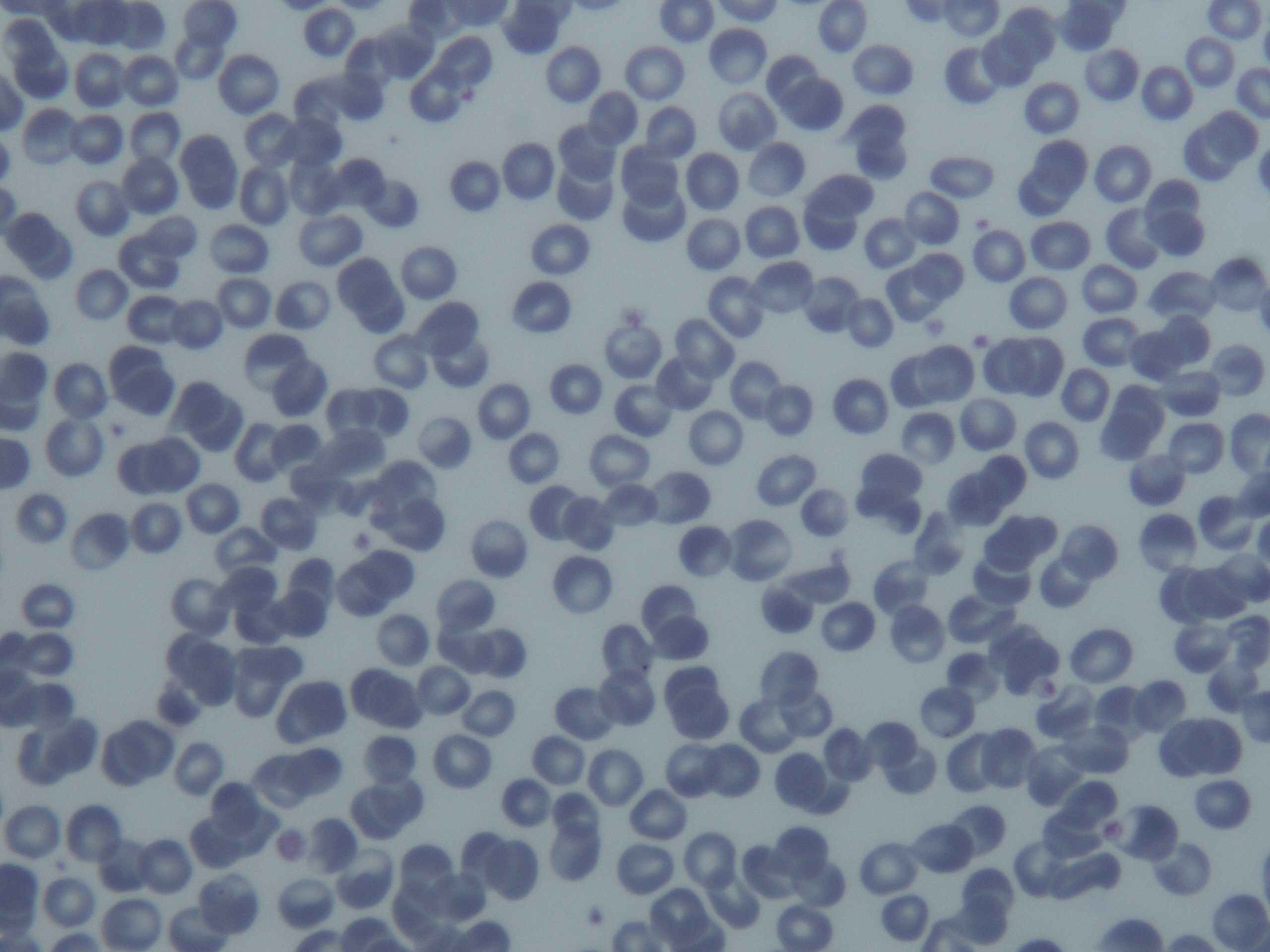


**Changes in erythrocyte morphology properties resulting from storage and ageing.** Microscopic examination of freshly drawn blood from a healthy donor blood smear (A). Detection of a variety of morphological alterations in red blood cells induced in the cell membrane as a consequence of accelerated ageing for a period of three days. Red blood cells acquired morphologic features of spiculated spherocytes (spheroechinocytes) (B). EDTA anticoagulated whole blood was stored at room temperature.

Supplementary Figure III

B)


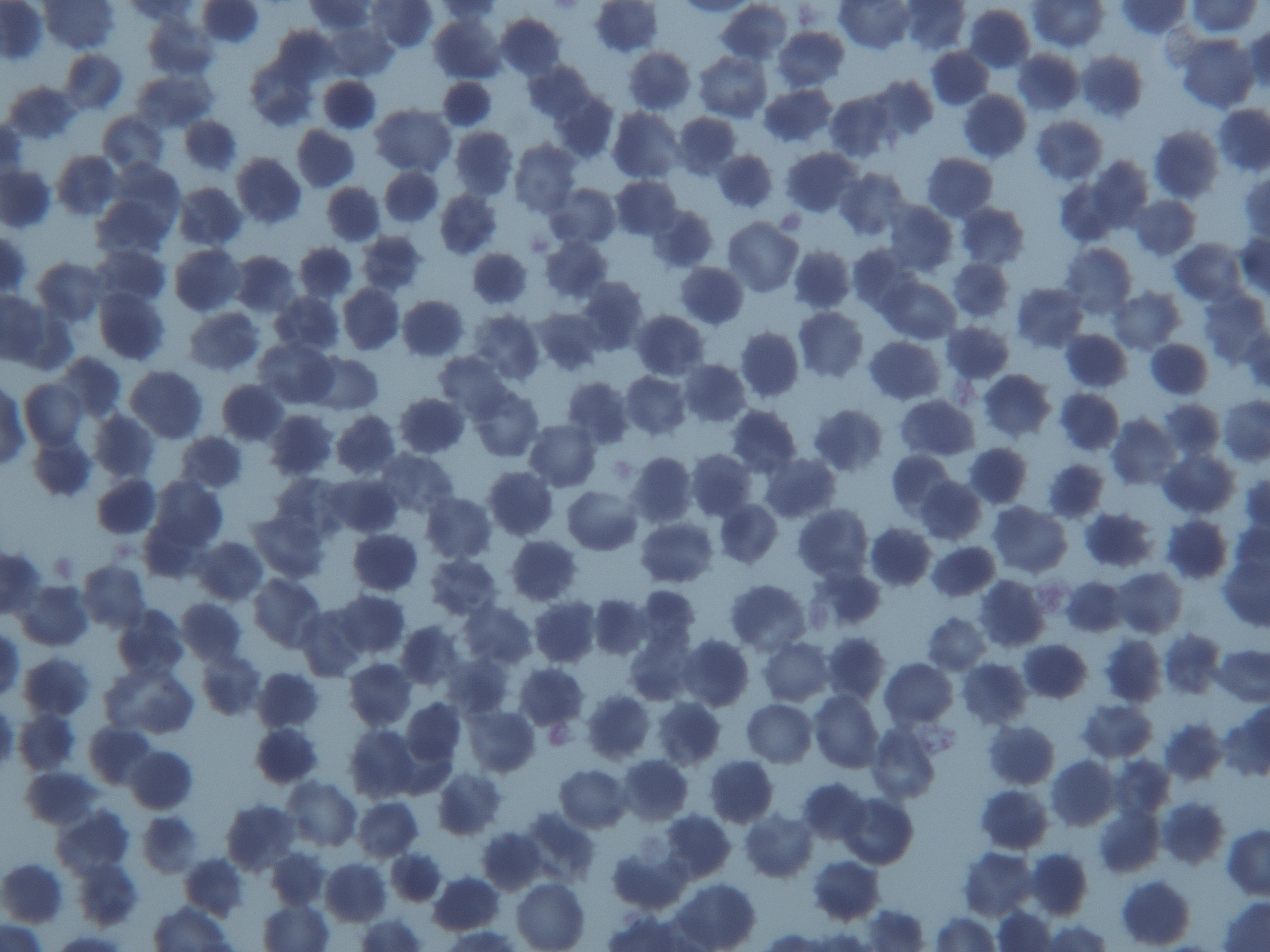


**Changes in erythrocyte morphology properties resulting from storage and ageing.** Microscopic examination of freshly drawn blood from a healthy donor blood smear (A). Detection of a variety of morphological alterations in red blood cells induced in the cell membrane as a consequence of accelerated ageing for a period of three days. Red blood cells acquired morphologic features of spiculated spherocytes (spheroechinocytes) (B). EDTA anticoagulated whole blood was stored at room temperature.

Supplementary Figure IV


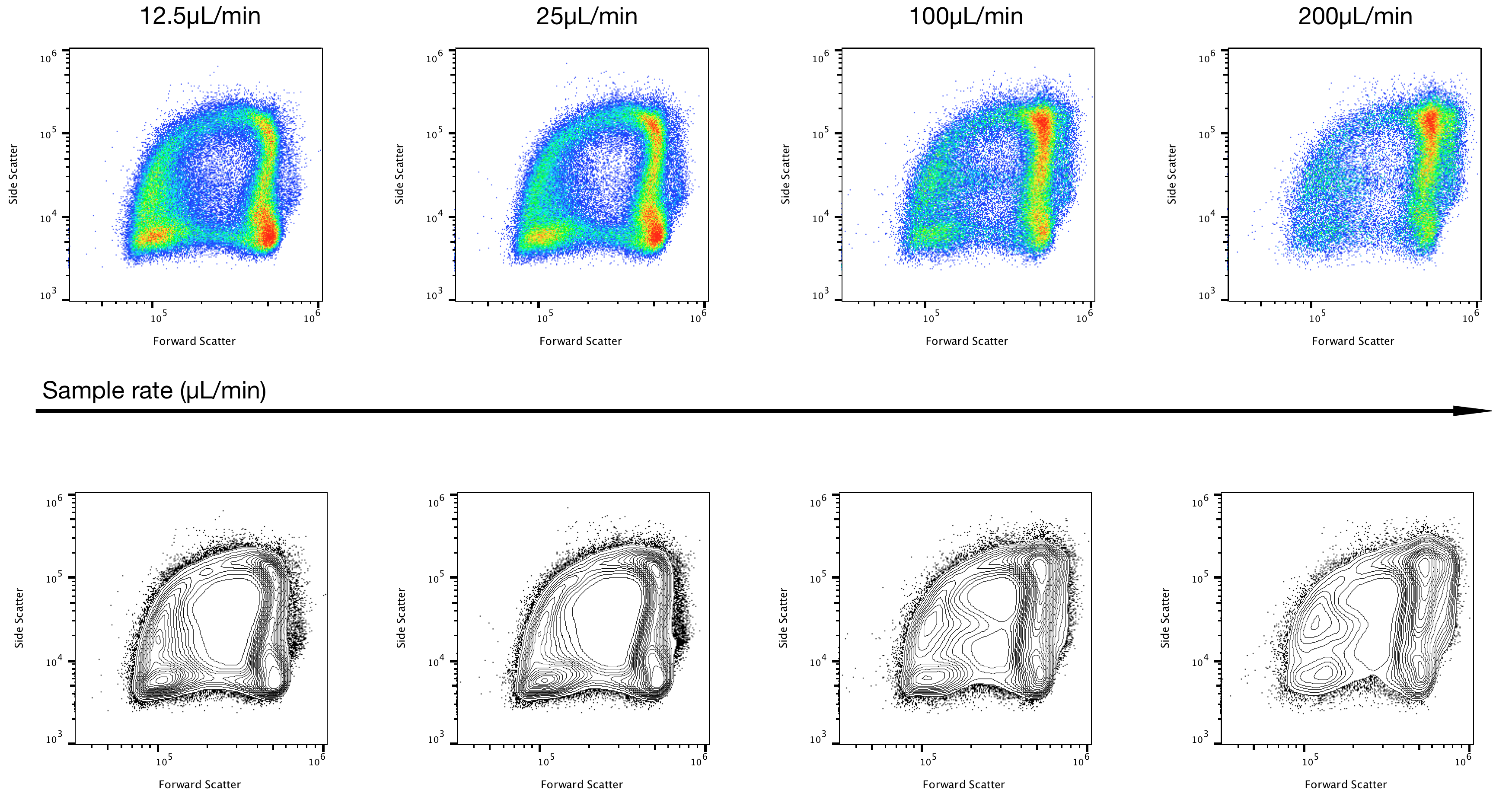


**Comparative effect of the flow speed using unlysed diluted blood.** Representative distribution of blood cells showing red blood cells (RBCs) at 12.5, 25, 100 and 200µL/min. Forward scatter vs. side scatter dotplots (upper row) and contour plots (lower row) display well-defined characteristic arch-shaped populations of erythrocytes at 12.5 and 25µL/min, and degraded scatter as 100 and 200µL/min.

Supplementary Figure V


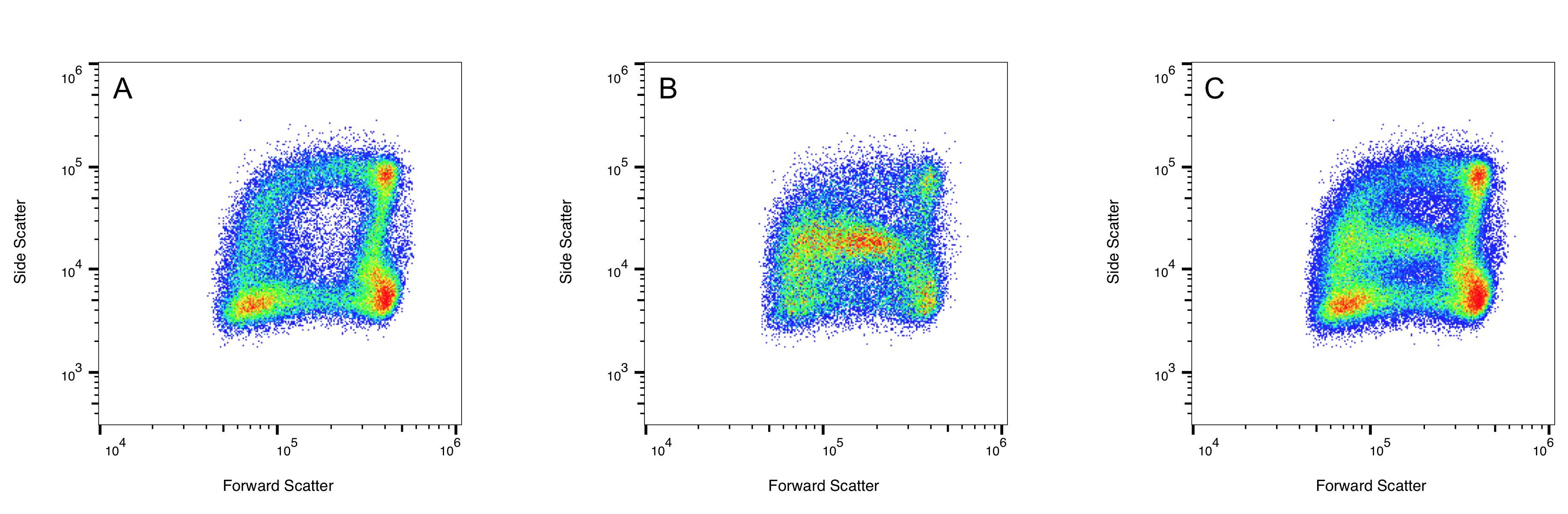


**Combined populations from different files into one FCS data file, displaying normocytes, spherocytes and the overall population.** Concatenated sample data obtained from normocytes (n=10) and spherocytes (n=10). All FCS files (n=20) were electronically concatenated into one FCS file and randomly downsampled to 100,000 events using FlowJo v10. For comparative analysis, the new concatenated file, was used to display normocytes (A), and spherocytes (B). The whole merged population was used to display a uniformly random sample of the overall population containing both normocytes and spherocytes (C).
